# Mumps Vaccination Policies and Epidemiological Consequences: A Comparative Review of the United States and Japan

**DOI:** 10.3390/epidemiologia7040095

**Published:** 2026-07-07

**Authors:** Naruhito Otani, Masaki Ohmuraya, Toshiomi Okuno

**Affiliations:** 1 Department of Genetics, Hyogo Medical University, Nishinomiya 663-8501, Japan; ohmuraya@hyo-med.ac.jp; 2 Department of Public Health, Hyogo Medical University, Nishinomiya 663-8501, Japan; 3 Department of Microbiology, Hyogo Medical University, Nishinomiya 663-8501, Japan; tmokuno@hyo-med.ac.jp

**Keywords:** mumps, mumps vaccine, waning immunity, MMR vaccine, adverse event following immunization (AEFI)

## Abstract

Mumps remains a public health challenge worldwide despite being a vaccine-preventable disease. This review outlines the historical development and contrasting mumps vaccination policies in the United States and Japan and their epidemiological consequences. In the United States, where the two-dose measles, mumps, and rubella (MMR) vaccine is routinely administered, the incidence of mumps has markedly decreased. Recently, however, sporadic outbreaks have emerged, driven by breakthrough infections among vaccinated individuals. These resurgences are driven largely by waning immunity, coupled with antigenic divergence between the vaccine strain and circulating wild-type viruses. In contrast, Japan vaccination the MMR vaccine was licensed in 2026 after being suspended in 1993 owing to concerns over aseptic meningitis as an adverse event following immunization but mumps vaccination remains voluntary. This has resulted in low vaccination coverage, a lack of herd immunity, and recurrent epidemics. This review also highlights the disease-modifying effect of mumps vaccination, including a reduced risk of complications such as meningitis and orchitis, even in cases of breakthrough infection. In Japan, irreversible sensorineural hearing loss (mumps deafness), continues to occur even following subclinical infection. To prevent these complications, continuous molecular surveillance, improved vaccine safety, and reintroduction of routine two-dose mumps vaccination in Japan are essential.

## 1. Introduction

Mumps is an acute viral infectious disease caused by the mumps virus, which belongs to the genus *Rubulavirus* in the family *Paramyxoviridae*. It is transmitted primarily through respiratory droplets and direct contact. After an incubation period of 2–3 weeks, it manifests primarily as non-suppurative swelling of the parotid and submandibular glands, accompanied by fever. Although the prognosis is generally favorable, the virus can spread to various organs, and can cause severe complications such as aseptic meningitis, postpubertal orchitis and oophoritis, pancreatitis, and irreversible sensorineural hearing loss (mumps deafness) [1,2].

Mumps is inherently a vaccine-preventable disease. In many countries, including the United States, a routine two-dose immunization schedule with the measles, mumps, and rubella (MMR) vaccine has been implemented, leading to a marked reduction in disease incidence compared with that in the pre-vaccine era. Recently, however, sporadic outbreaks driven by breakthrough infections among vaccinated individuals have emerged as a new public health challenge, even in populations with high vaccination coverage, largely attributable to waning immunity over time [2,3,4].

The situation is different in Japan. Although routine immunization with the MMR vaccine was introduced in 1989, aseptic meningitis caused by the mumps vaccine strain became a public health issue, forcing the suspension of the MMR vaccine in 1993 [5]. Since then, mumps vaccination has been voluntary in Japan. Consequently, vaccination coverage remains below the threshold for herd immunity, resulting in periodic large-scale epidemics unusual for a developed country. Japan is currently in a state of a “vaccine gap,” where severe but preventable complications of mumps continue to occur [1].

This review outlines the historical development of mumps vaccines and overviews the evolution of immunization policies and epidemiological trends in the United States and Japan. Furthermore, it summarizes recent knowledge regarding immunological mechanisms and diagnostics, elucidates the role of vaccines in mitigating complications, and comprehensively discusses current challenges and future perspectives in mumps control.

## 2. History of Mumps Vaccines

The initial development of mumps vaccines focused on inactivated vaccines; however, as they did not provide a sufficient level of protection, the focus shifted to the development of live, attenuated vaccines. In the former Soviet Union, the Leningrad-3 (L-3) strain was developed as a live attenuated mumps vaccine in 1956 [6] and was widely used. This lineage was subsequently improved and established as the L-Zagreb strain, which is still used in some regions [7].

In the United States, the Jeryl Lynn strain was developed and generated as the vaccine strain in 1965 [8]. This vaccine strain was licensed in 1967 and remains the only approved mumps vaccine strain in the United States. Furthermore, the Jeryl Lynn strain is widely used globally [8,9].

In Japan, research and development of live, attenuated mumps vaccines started in the 1970s using the Urabe, Hoshino, and Torii domestic strains [10,11,12]. Urabe and Hoshino strain vaccines were approved in 1980, and a Torii strain vaccine was approved in 1982. Currently, the two types of monovalent mumps vaccines manufactured and available in Japan use the Torii and Hoshino strains.

## 3. Evolution of Mumps Vaccination Policies

The historical mumps vaccination policy milestones in the United States and Japan is summarized in Table 1.

### 3.1. United States

In the United States, the mumps vaccine was first licensed in 1967, and since 1971 has been administered as part of the MMR vaccine [13]. The Advisory Committee on Immunization Practices (ACIP) recommended inclusion of the MMR vaccine in the national immunization schedule as a single dose in 1971 and later revised the recommendation to a two-dose regimen in 1989 [14].

Although this 1989 revision was primarily intended to provide two doses of measles vaccine to all children [14], because the measles vaccine is typically administered as the MMR vaccine in the United States, it consequently established a system in which most children also received two doses of the mumps vaccine.

### 3.2. Japan

In Japan, voluntary immunization with the monovalent mumps vaccine began in 1981, followed by the introduction of the MMR vaccine as a routine immunization in 1989 [5]. Notably, the mumps component of the MMR vaccine adopted at that time was a “unified-strain” determined by the national government and produced by local manufacturers, using the Urabe strain as its mumps component. However, the occurrence of aseptic meningitis caused by the mumps vaccine strain included in the MMR vaccine became a widespread public health concern, leading to the discontinuation of the MMR vaccine in April 1993. Since then, mumps vaccination in Japan reverted to voluntary (non-routine) immunization using the monovalent vaccine [1].

## 4. Epidemiological Trends and Mumps Surveillance Systems

### 4.1. United States

Before the introduction of the vaccine in the United States, mumps was an infectious disease that primarily affected children. A survey by the U.S. Public Health Service (1960–1964) reported that 92% of all cases occurred in children aged under 15 years [15].

Following the licensure of the live attenuated mumps vaccine in 1967, mumps was designated as a nationally notifiable disease in 1968. In 1968, 152,209 cases of mumps were reported to the U.S. Centers for Disease Control and Prevention (CDC). However, with the widespread implementation of vaccination, the number of reported cases declined markedly, and dropped to 2886 cases (1.2 per 100,000 population) in 1985, a 98.1% decrease compared with the number of cases reported in 1968 [16].

An age-specific analysis of mumps incidence in 1967–1976, in the period immediately following vaccine licensure, revealed that the incidence rate was highest among children aged 5–9 years, followed by children under 5 years, with these two groups accounting for over 70% of all cases [16]. Subsequently, in an analysis of cases reported from 1988 to 1993, the major affected age groups shifted to 5–14 years (52%) and 15 years and older (36%), and the incidence rate dropped from 2.0 per 100,000 in 1988 to 0.7 per 100,000 in 1993 [17]. By 1993, the number of reported cases reached a historic low in the National Notifiable Diseases Surveillance System with only 1692 cases reported, a reduction of approximately 99% compared with that in 1968 [17].

Although the number of reported cases has been maintained at low levels owing to the establishment of the two-dose MMR vaccine regimen, sporadic resurgences (outbreaks) have occurred since the early 2000s. Notably, in 2006, a large, multi-state outbreak occurred primarily among university student populations, with over 6500 reported cases, the majority of whom had a history of receiving two doses of MMR vaccine [3]. Furthermore, between January 2016 and June 2017, 150 outbreaks and a total of 9200 cases were reported, confirming transmission in group settings such as households, schools, universities, and sports teams [18].

These events suggest that although maintaining high vaccination coverage contributes to long-term disease control in the United States, sporadic resurgence can occur, driven by waning immunity over time and intense exposure in crowded living environments.

### 4.2. Japan

The epidemiological trends of mumps in Japan have been closely linked to changes in vaccination policies. The evaluation of long-term national trends is based on sentinel surveillance using the National Epidemiological Surveillance of Infectious Diseases (NESID) database. Mumps is classified as a Category V infectious disease (subject to sentinel surveillance) under the Infectious Diseases Control Law, and case numbers are reported weekly by approximately 3000 pediatric sentinel surveillance sites nationwide [1]. This sentinel surveillance system allows for the monitoring of epidemic trends using standardized, regionally representative data [1,19].

The mumps vaccine was introduced as a voluntary vaccination in 1981. Prior to the introduction of mumps vaccine, a unified national surveillance system was not in place, so the national trends in mumps incidence are unknown. Consequently, the evaluation of long-term epidemiological trends in Japan is based primarily on analysis of sentinel surveillance data that has accumulated since sentinel surveillance was introduced in 1982 [20].

After the initiation of sentinel surveillance, outbreaks generally occurred approximately once every 3 to 4 years during the 1980s and early 1990s [20]. After the introduction of the MMR vaccine in April 1989, the number of reported cases to dropped to low levels between 1990 and the first half of 1993, with the number of cases per sentinel site remaining below 1.0 [20]. However, after the discontinuation of the MMR vaccine in April 1993, periodic large-scale outbreaks re-emerged, and have continued since the early 2000s [1,19].

According to annual data since 1999, large-scale epidemics were confirmed with 132,877 cases (44.62 per sentinel site) in 2000 and 254,711 cases (84.37 per sentinel site) in 2001 [19]. Since then, the number of outbreaks has continued to fluctuate. Although the number of cases per sentinel site has remained relatively low in recent years (15,153 cases/4.80 per site in 2019; 8073 cases/2.56 per site in 2020; 4933 cases/1.57 per site in 2022; and 6864 cases/2.19 per site in 2023), complete control has not been achieved [19].

Because sentinel surveillance is based primarily on cases reported in pediatric sentinel clinics, the number of adult cases may be underestimated [1]. Therefore, when evaluating mumps epidemiology in Japan, the data should be carefully interpreted, focusing on long-term trends based on sentinel surveillance reports while considering the historical policy changes (i.e., the introduction and discontinuation of the MMR vaccine) and the current institutional background of voluntary vaccination policy [1,5,19].

## 5. Vaccination Coverage

### 5.1. United States

In the U.S., vaccination with the MMR vaccine is mandated for school entry. This has maintained high levels of two-dose coverage for many years. In the 2015–2016 school year, the reported two-dose MMR vaccination coverage was reported as 94.6% among children attending kindergarten [20], and 90.7% among adolescents aged 13–17 years [21].

However, vaccination coverage has declined in recent years. According to CDC reports, the two-dose MMR vaccination coverage was 92.7% among children attending kindergarten in the 2023–2024 school year, remaining below the public health target of 95% [22]. This decline was attributed to restricted healthcare access and delays in routine immunizations during the COVID-19 pandemic [22].

### 5.2. Japan

In contrast to the United States, mumps vaccination remains voluntary in Japan. A 2016 survey found that mumps vaccination coverage among children was only 30–40%, and the seropositivity rate was approximately 70% [1]. Furthermore, even in the immunization surveillance data from 2024, the vaccination coverage has remained largely unchanged [23]. The coverage falls short of the estimated threshold of 75–93% necessary for herd immunity, and the lack of herd immunity is the primary reason for the recurrent outbreaks in the country [1].

Furthermore, although the number of reported cases has remained low since the COVID-19 pandemic [19,24], this is likely a temporary effect due to reduced exposure opportunities associated with infection control measures. Strong concern has been expressed about the possibility of resurgent outbreaks owing to a build-up in the number of susceptible individuals [24,25].

## 6. Immune Response and Vaccine Effectiveness

### 6.1. Mechanisms and Durability of Immunity

Immunity against mumps is induced by either vaccination or natural infection and involves both humoral and cellular immunity. Following MMR vaccination, antibodies tend to persist for a long period: mumps antibodies are detectable in approximately 70–99% of vaccinees 10 years after the initial dose [2]. Furthermore, mumps-specific T-lymphocyte responses are detectable in approximately 70% of adults who received the vaccine in childhood, indicating that cellular immunity is also maintained, although vaccine-induced cellular immunity is slightly lower than that following natural infection (approximately 80%) [2].

Clinical and public health criteria for confirming mumps immunity include documented records of appropriate vaccination, serological confirmation of immunity (detection of mumps-specific immunoglobulin G [IgG] antibodies), record of past infection, or being born before 1957 (according to U.S. criteria) [2].

### 6.2. Protective Effects and Limitations of Vaccines

The mumps vaccine has been shown to be effective at preventing disease onset and severe illness. The effectiveness in preventing disease is approximately 70–80% following a single dose of MMR vaccine and approximately 80–90% following two doses of MMR vaccine, indicating that a second dose improves the level of protection [2,4].

However, vaccination does not provide complete sterilizing immunity, and outbreaks driven by breakthrough infections occur periodically even in populations that have completed the two-dose series [4]. This phenomenon is largely attributed to waning immunity and the force of infection. Long-term follow-up studies have shown that although antibodies are still detectable in 74–95% of individuals 12 years after the second dose of vaccine, antibody titers decline over time [2]. Observations from outbreaks suggest that the risk of developing the disease increases with the number of years elapsed since received the second dose of vaccine [4]. The high variability of T cells, including their capacity to revert to a naïve-like state, presents an inherent challenge to maintaining stable, durable, long-term immune memory [26]. In close-contact settings such as university dormitories and sports teams, vaccinated individuals can acquire infection if they are exposed to a high viral load, even if vaccine-induced immunity is maintained [2,4].

When vaccinated individuals experience breakthrough infections, their symptoms generally remain mild, and the risk of complications is reduced. However, vaccinated individuals with mildly symptomatic or asymptomatic infection can contribute to transmission within the community [2].

### 6.3. Impact of Genotype Mismatch on Breakthrough Infections

Another critical factor that may have contributed to recent surges in breakthrough infections among vaccinated individuals is the antigenic divergence between the vaccine strains and the circulating wild-type viruses, referred to as “genotype mismatch” [27]. Mumps viruses are currently classified into 12 genotypes (A through N, excluding E and M). The live attenuated vaccines most widely used worldwide, including the Jeryl Lynn strain used in the United States and the Torii and Hoshino strains used in Japan, all belong to Genotype A [28].

However, global surveillance data has shown that in recent decades infections caused by Genotype A wild-type viruses have declined to very low levels and Genotype G has emerged as the globally dominant lineage, responsible for the majority of the large-scale outbreaks in North America, Europe, and Japan [28]. Other genotypes, including Genotypes F and H, have been reported sporadically in different regions.

Immunological studies have demonstrated that although Genotype A-derived vaccines induce cross-neutralizing antibodies against non-A genotypes, the neutralization titers against the currently circulating Genotype G viruses are substantially lower than those against the homologous Genotype A vaccine strains [29]. This reduced cross-neutralization capacity suggests that the vaccine-induced humoral immunity may not be sufficient to block infection when an individual is exposed to a high viral load of the heterologous Genotype G wild-type virus [29].

Although waning immunity is thought to be the primary driver of mumps resurgence, this antigenic mismatch contributes to transmission in highly vaccinated populations in group settings [28]. Therefore, continuous molecular surveillance of circulating mumps virus genotypes is necessary and consideration should be given to updating vaccine strains to reflect circulating genotypes for effective outbreak control [28].

## 7. Basic Strategies for Outbreak Control

The basic strategies for controlling a mumps outbreak are the early detection and isolation of symptomatic individuals, coupled with the rapid identification and management of contacts [30]. To prevent secondary transmission from symptomatic individuals, an isolation period of 5 days from the onset of parotitis is recommended [30].

Catch-up vaccination for unvaccinated individuals and verification of vaccination histories are also critically important because even those who have completed the two-dose series are at risk of disease owing to waning immunity over time [4]. In high-exposure group environments, administering a third dose of MMR vaccine as a booster and mass vaccination campaigns have been shown to contribute to outbreak control [3,4,30]. However, administering a third dose of MMR vaccine is currently recommended only as a targeted intervention during outbreaks, and is not recommended for routine immunization during non-outbreak periods [3,4,30].

## 8. Diagnosis and Laboratory Testing

The diagnosis of mumps is typically made clinically based on the presence of parotitis. However, in settings where disease incidence is low, laboratory testing is essential for case confirmation because parotitis can also be caused by other viral infections [31].

Serological diagnosis is conventionally performed by detecting immunoglobulin M (IgM) antibodies in acute-phase serum or by demonstrating a significant rise in IgG antibody titers between paired acute and convalescent sera. However, although IgM is readily detectable in unvaccinated individuals, the IgM detection rate is 15% or less in individuals who have received two doses of vaccine [32]. Furthermore, in vaccinated individuals, IgG antibody titers are often already elevated in the early stages of disease, making demonstration of a clinically significant increase in titers using paired sera difficult [32].

Virological diagnostic methods include tests based on reverse transcription-polymerase chain reaction (RT-PCR) and viral culture, with RT-PCR now widely used to confirm the diagnosis [31,33]. In Japan, however, RT-PCR testing for mumps is not covered by the national health insurance system and is therefore not widely used in clinical practice.

The success rate of viral detection by RT-PCR depends on the timing of sample collection, sample handling, target genes, and the patient’s vaccination history. In a study using oropharyngeal swabs collected on the day of parotitis onset from presumably unvaccinated children, mumps virus RNA was detected in 85% of clinical specimens [33]. Similar results have been reported for recipients of a single vaccine dose, but the detection rate drops to approximately 30% in samples from individuals who have received two doses of vaccine [32].

The positivity rate of RT-PCR has been shown to be substantially higher when swabs are collected soon after symptom onset, and this is particularly evident among patients with a history of vaccination. In one study of patients with a history of receiving one or two doses of a mumps-containing vaccine, 68% of samples collected within 2 days of onset were RT-PCR positive, whereas the positivity rate was 21% for samples collected 3 or more days after the onset [32]. In contrast, infection remained detectable for longer in unvaccinated individuals (55% in samples collected within 2 days and 50% in samples collected on day 3 or later). These findings suggest that the duration of viral shedding may be shortened in vaccinated individuals although current evidence is conflicting [32].

## 9. Complications and Disease-Modifying Effects of Vaccines

Multiple epidemiological studies suggest that mumps vaccination attenuates disease severity and reduces the risk of complications during breakthrough infections. This effect of reducing the incidence of complications is dose-dependent and increases with the number of doses of vaccine received. According to an epidemiological analysis of confirmed outbreaks of mumps in England and Wales between 2002 and 2006 [34], using unvaccinated individuals as the baseline (odds ratio 1.0), the unadjusted odds ratio for developing meningitis decreased to 0.31 in individuals who had received one dose of vaccine and 0.22 for those who had received two doses of vaccine. Similarly, for orchitis, the odds ratios were 0.44 for those who had received one dose of vaccine and 0.17 for those who had received two doses of vaccine. Furthermore, vaccination was associated with a dose-dependent decrease in the risk of requiring hospitalization [34]. Similar trends have been reported in investigations of large-scale epidemics in the United States from 2009 to 2010. In an analysis of male patients aged 12 years and older, the 11% of unvaccinated individuals, but only 4% of those who had received two doses of vaccine developed orchitis as a complication [35]. Other studies have also reported that vaccination (especially two doses) contributes to a reduction in the overall incidence of complications and the risk of hospitalization [36]. These findings suggest that even when mumps vaccination does not prevent infection, it reduces the incidence of complications such as meningitis and orchitis.

## 10. Mumps Deafness (Sensorineural Hearing Loss) in Japan

One of the most severe complications of mumps is irreversible sensorineural hearing loss (mumps deafness). A nationwide survey conducted by the Oto-Rhino-Laryngological Society of Japan during the 2015–2016 epidemic revealed at least 359 cases of onset of acute sensorineural hearing loss during the 2-year epidemic period [37]. The age distribution of cases of mumps showed a bimodal peak among children aged 3–15 years and among adults in their 30s, who are presumed to have contracted infection from their children. Furthermore, 26.5% of all cases of hearing loss were in individuals with subclinical infections without parotid swelling, indicating that mumps can cause sudden hearing loss even in the absence of specific signs of disease [37].

The greatest challenge of mumps deafness is its extremely poor response to treatment. In the same survey, only 5.0% of individuals with hearing loss regained their hearing, even with treatments such as steroids, and 27% experienced a deterioration in hearing during the course of treatment. Ultimately, 90.7% of cases of unilateral hearing loss and 80% of cases of bilateral hearing loss led to permanent severe hearing loss, and many participants required cochlear implants or hearing aids [37].

These results clearly demonstrate that once mumps deafness develops, restoring hearing is extremely difficult even with modern medicine, and currently the only effective countermeasure is primary prevention of infection through vaccination. The high incidence of preventable profound hearing loss in Japan owing to low vaccination coverage is a critical public health concern.

## 11. Future Prospects and Challenges for Mumps Control in Japan

A noteworthy advancement is the approval of Daiichi Sankyo’s MMR vaccine, Mimrit, in Japan in May 2026. This vaccine uses the Jeryl Lynn strain, which has been used extensively globally [8,9]. The approval of Mimrit necessitates a careful assessment of its long-term epidemiological and public health impact. Improved mumps vaccination coverage will reduce the circulation of wild-type mumps virus, leading to the loss of “natural boosting”—the periodic immune reinforcement provided by re-exposure to the wild virus. As vaccine-induced immunity wanes over time [2,4], susceptibility to mumps could eventually shift toward older age groups following an initial period of low incidence. Therefore, ensuring high coverage with two doses of mumps virus-containing vaccine for children is essential to establish herd immunity.

Furthermore, transition to routine vaccination with MMR vaccine is essential in order to harmonize the relief systems for adverse events following immunization (AEFI). Currently, measles and rubella vaccination are classified as “routine,” whereas mumps vaccination remains “voluntary,” creating a discrepancy in the legal framework for compensation for AEFI. Enactment of legislation to integrate mumps vaccination into the routine vaccination schedule along with measles and rubella vaccination is essential to resolve this administrative and legal inconsistency. Additionally, the adoption of the Jeryl Lynn strain, which has been used extensively worldwide, rather than the local Japanese Torii and Hoshino strains used in mumps-only vaccines, is a key strategy for addressing public anxieties regarding complications.

Regarding the pool of susceptible adults, Japan’s history with the measles-rubella (MR) vaccine offers an important lesson. During the period when rubella vaccination targeted females only, a large number of cases of rubella were reported among unvaccinated males [38], demonstrating that targeting specific demographic groups defined based on age or sex can have negative repercussions for excluded demographic groups. Therefore, once routine mumps vaccination is introduced, rigorous nationwide serosurveillance and monitoring of epidemiological trends will be necessary to identify vulnerable demographic groups. Based on these results, targeted catch-up programs should be considered.

## 12. Conclusions

This review shows that the mumps vaccination is a vital public health tool that markedly reduces the incidence of the disease and its severe complications. Epidemiological evidence suggests that the risk of complications decreases as the number of doses of vaccine increases and supports the use of vaccination, even though vaccination does not completely prevent breakthrough infections. However, maintaining complete sterilizing immunity over a long period through vaccination is challenging, and the occurrence of breakthrough infections—driven by waning immunity over time and high exposure intensity in crowded environments—has emerged as a barrier to mumps control.

In the United States where vaccination coverage is high, administering a third (booster) dose during outbreaks and continuous surveillance of antigenic differences between circulating wild-type strains and vaccine strains is effective for disease control.

Conversely, in Japan where mumps vaccination is currently voluntary and MMR vaccine has only recently been licensed after being unavailable for 33 years, the challenges are far greater. Low vaccination coverage has led to a lack of herd immunity and recurrent epidemics, and “mumps deafness” is an unresolved public health concern. To minimize the negative health effects of mumps in Japan, a two-dose mumps vaccination schedule should be adopted as part of routine immunization, and public trust in the safety of the MMR vaccine needs to be established. However, even with improved pediatric mumps vaccination coverage, mumps outbreaks are likely to continue to occur for the foreseeable future owing to large numbers of susceptible unvaccinated adults, as observed with measles and rubella in Japan during the rubella and measles elimination phases.

## Figures and Tables

**Table 1:** Timeline of major mumps vaccination policy milestones in Japan and the United States.

Year	Japan	USA
1967	—	Vaccine approval
1971	—	Routine one-dose vaccination started
1980	Vaccine approval	—
1981	Voluntary ^1^ one-dose vaccination started	—
1989	Routine MMR started	Routine two-dose vaccination started
1993	MMR program suspended (reverted to voluntary)	—
2026	Mimrit MMR vaccine licensed	—

^1^ In Japan, “voluntary” vaccination refers to non-routine immunizations that are not legally mandated and generally require out-of-pocket payment, leading to lower coverage than in countries with routine programs. MMR, measles, mumps, and rubella.

## Data Availability

No new data were created or analyzed in this study. Data sharing is not applicable to this article.
